# Failure behavior of nylon products for red phosphorus flame retardant electrical connectors

**DOI:** 10.1039/c9ra04027g

**Published:** 2019-08-12

**Authors:** Zhi Chen, Jianxin Du, Xiangmei Li, Zhili Xie, Yan Wang, Huiping Wang, Jiechang Zheng, Rongjie Yang

**Affiliations:** School of Materials Science and Engineering, Beijing Institute of Technology, National Engineering Research Centre of Flame Retardant Material Beijing 100081 China bjlglxm@bit.edu.cn; SAMR Defective Product Administrative Centre Beijing 100088 China

## Abstract

The failure behavior of red phosphorus flame retardant electrical connectors was investigated by their thermal degradation, changes of surface morphology and elements under an accelerating environmental experiment. The results showed that the nylon electrical connector degraded and cracks were generated on the surface. The temperature of maximum weight loss rate was advanced in the thermogravimetric analysis and the third weight loss peaks appeared after 28 days. The relative atomic content of O and P increased from 0 day to 28 days. The chemical environment of C changed. Partially, C–C bonds broke and became C–O bonds. The release of phosphine increased and this further oxidized to an acid with P=O and P–O groups in a warm and humid environment.

## Abstract 2

The failure mechanism of red phosphorus retardant PA66 electrical connectors is investigated.

## Graphical abstract

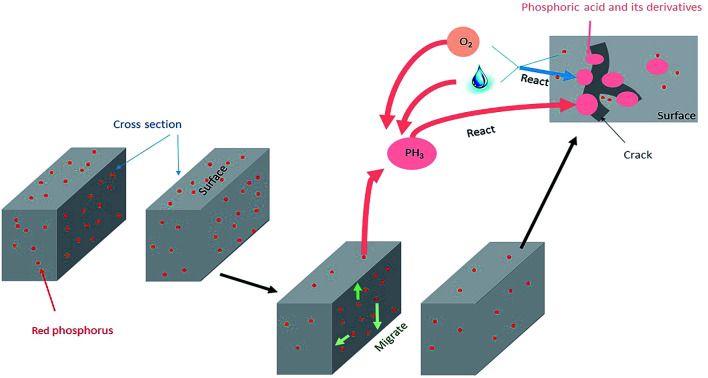

## Introduction

Electrical connectors are indispensable electronic components of electronic and electrical systems.^1^ They act as “bridges” in the circuit to realize the transmission of signals and electrical energy.^2^ Their quality has a huge impact on the performance and quality of the products.^3^ With maximum output and various types, polyamide is the most widely used engineering plastic.^4–7^ And, PA 66 has good mechanical properties and electrical properties, such as wear resistance, oil resistance, self-lubrication and acid and alkali resistance.^8,9^ Therefore, PA66 has broad application potentials for electrical and electronic connectors. The limiting oxygen index value of PA66 is 22.5%,^10^ reaching V-2 classifications in the UL-94 texts.^11^ However, the electrical connector has higher requirements for flame retardancy and the addition of glass fiber further reduces the flame retardancy of PA with “wick effect”.^12^ The use of electrical connectors can cause irreversible damage to electronic and electrical products, and even further threaten human's life. Therefore, the use of flame retardants becomes very important.

With low cost, low loaded amount, environmental friendliness and little influence on mechanical properties, red phosphorus is a kind of excellent flame retardant.^13–15^ It is widely used as a flame retardant material for electrical and electronic connectors in the industrial production. Red phosphorus would form a liquid film and a carbon layer, which will affect the kinetics of the thermal decomposition process of PA 66, so that achieve a flame retardant effect.^16,17^ The glass fiber reinforced PA 66 with 6–8% red phosphorus would reach V-0 level in the UL-94 test.^18^

However, phosphine is released during usage for red phosphorus and further produces phosphoric acid derivatives in a warm and humid environment,^19^ which causes damage to the performance of the connector and induces combustion. The reliability risks of using red phosphorus as a flame retardant material in encapsulated microcircuits was discussed. The field failure rate has been studied to drop from approximately 5000 ppm to 500 ppm when the maximum particle diameter is reduced from 180 to 150 μm for epoxy resin. The oxygen-containing phosphorus acids are corrosive and can alter the physical and electrical characteristics of the polymer composites.^20^ Michael Pecht considered these acids and ions generated could induce electro-chemical migration, causing short circuits in the electronic device encapsulation.^21^ PA 66 can be used as wire and cable, switch socket, relay, connector, *etc.*, with red phosphorus as a common flame retardant. Among them, Panasonic refrigerator and Amway connector caused fire accidents. However, the failure mechanism of red phosphorus flame retardant nylon products has not been systematically studied so far.

## Experimental

### Temperature and humidity environment experiment

**Fig. 1:**
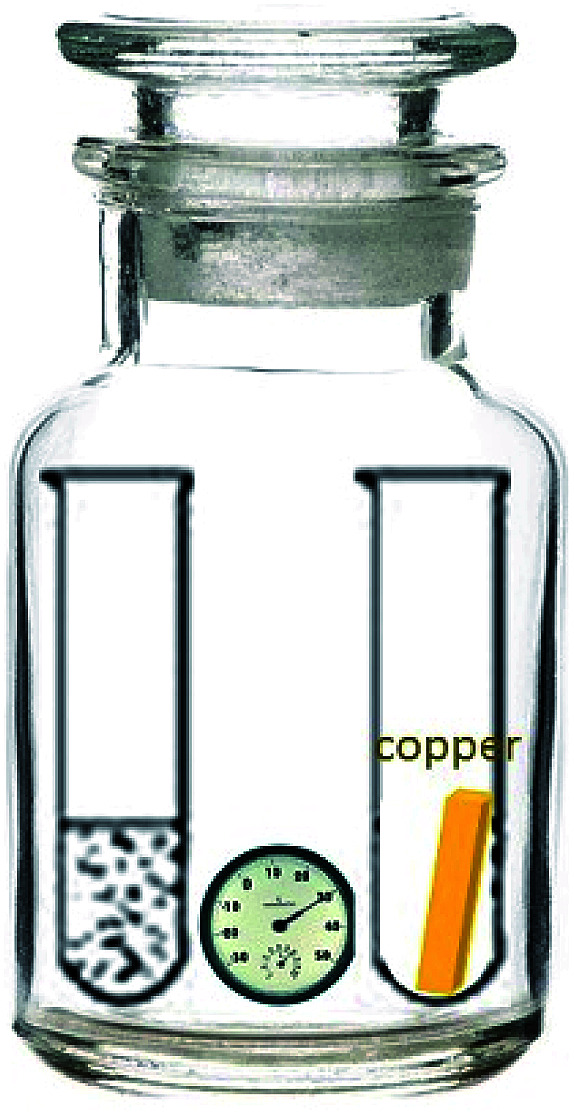
Schematic diagram of temperature and humidity environment experiment.

PA66 electrical connector products (hereinafter referred to as connectors), provided by Amway, was tested for aging at 80 °C, 75% RH. A 500 ml jar with the other 50 ml test tube containing distilled water was deposited in a drying oven at 80 °C, with a hygrometer showing the humidity inside at 75% RH (Fig. 1). The connector was disassembled after being cooled by liquid nitrogen. With copper sheets attached by a nylon cable tie, the connector was placed in the above-mentioned jar for 80 °C, 75% RH aging test. The products and copper sheets were characterized after 7 d, 14 d and 28 d respectively.

**Fig. 2:**
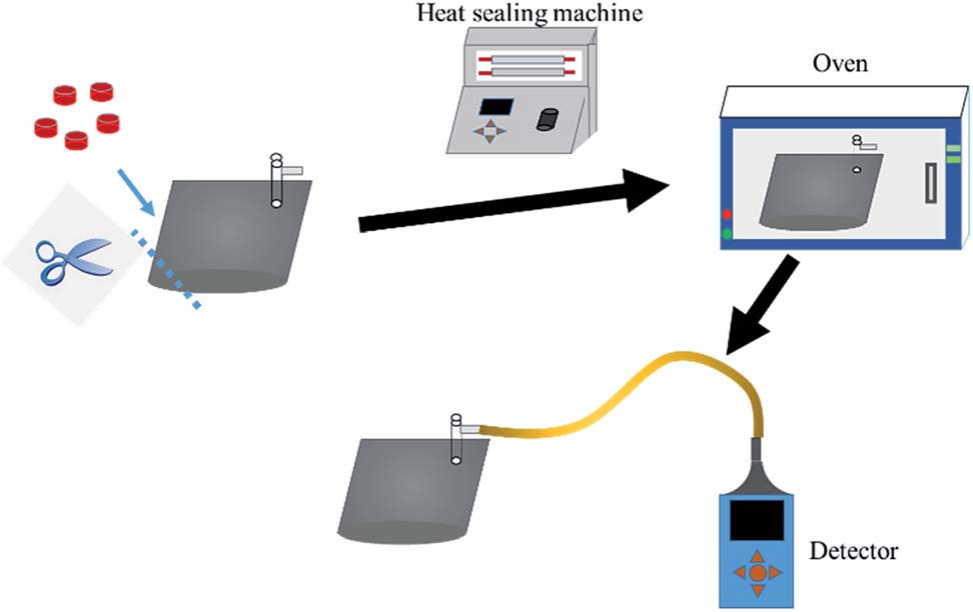
Schematic diagram of phosphine detection.

Phosphine released by red phosphorus would rapidly reacts with air and water to form an acid under high temperature and humidity. In order to determine phosphine release, 2 g product was deposited in a sealed and dried 500 ml aluminum foil gas collection bag (Fig. 2). After 7 d, 14 d and 21 d, the concentration of phosphine gas in the bag was determined by the BH-90A phosphine detector.

### Characterization

The morphology of connectors were observed by means of cold field-emission scanning electron microscopy (SEM, Hitachi S-4800, 10 kV), with products sprayed with gold to strengthen conductivity. The surface elements were analyzed by Energy Dispersive Spectroscopy (EDS) of the scanning electron microscopy.

A TGA instrument (TG 209 F1, NETZSCH, Germany) provided with an alumina crucible was used. The measurement was conducted in nitrogen (50 ml min^−1^) with a heating rate of 10 °C min^−1^ and samples of 2–3 mg from 40 to 800 °C. The experimental error was ±0.1% by weight.

The chemical structure of the connector was analyzed by a Fourier transform infrared spectrometer (Nicolet 6700, Thermo Electron Co., USA) over the wavenumbers range of 400–4000 cm^−1^.

X-ray photoelectron spectrometer (ESCALAB 250Xi, Thermo Fisher Scientific, USA) were performed on the products with a monochromated Al Kα radiation (*hν* = 1486.6 eV).

Inductively coupled plasma mass spectrometer (Agilent 7500ce, Agilent Technologies, USA) was used to measure the concentration of phosphorus of the substance formed on the surface of the copper sheets, dissolved in 10 ml dilute hydrochloric acid for 2–3 days.

## Results and discussion

### Thermogravimetric analysis

**Fig. 3:**
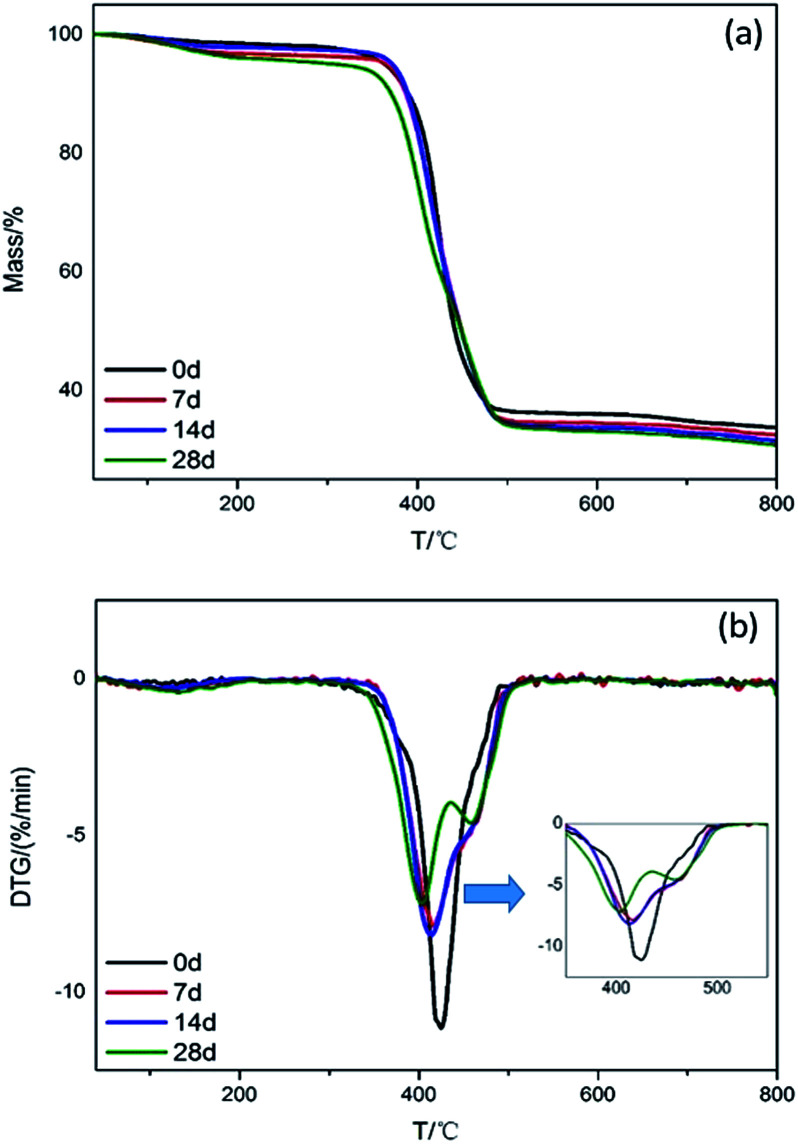
TGA and DTG curves of connectors in N_2_ atmosphere.

Thermal degradation behaviors of the connectors were investigated by TG and DTG, corresponding curves and data are presented in Fig. 3 and summarized in Table 1. As the environmental test progressing, the initial decomposition temperature (*T*_5%_, temperature based on 5% mass loss), the maximum mass loss rate (*R*_max_), temperature corresponding to the maximum mass loss rate (*T*_max_) and the value of char residues (CR) for connectors at 800 °C all reduced, indicating that the thermal stability of connectors exposed to high temperature and humidity environment diminished. The appearance of the mass loss rate peak for 28 days indicates that the connector produced a new component in the high temperature and humidity environment. Reduced CR indicated that the phosphine released from red phosphorus reacted with water to form various oxygen-containing, phosphorus-based acids, such as phosphoric acid, phosphorous acid, and hypophosphorus acid, and then partially transferred to the copper sheet by corrosion, resulting in a reduced char forming ability.

**Table 1:** Thermal decomposition data of connectors in N_2_

*T*/d	*T*_5% loss_ (°C)	*R*_max1_ (% min^−1^)/*T*_max1_ (°C)	*R*_max2_ (% min^−1^)/*T*_max2_ (°C)	*R*_max3_ (% min^−1^)/*T*_max3_ (°C)	CR (800 °C, N_2_)/%
0	363.6	0.20/129.3	11.15/424.8	—	33.71
7	365.5	0.38/129.5	7.9/416.5	—	32.47
14	372.1	0.32/108.4	8.2/413.6	—	31.52
28	306.1	0.44/131.7	7.19/403.6	4.6/458	30.78

### FTIR analysis

**Fig. 4:**
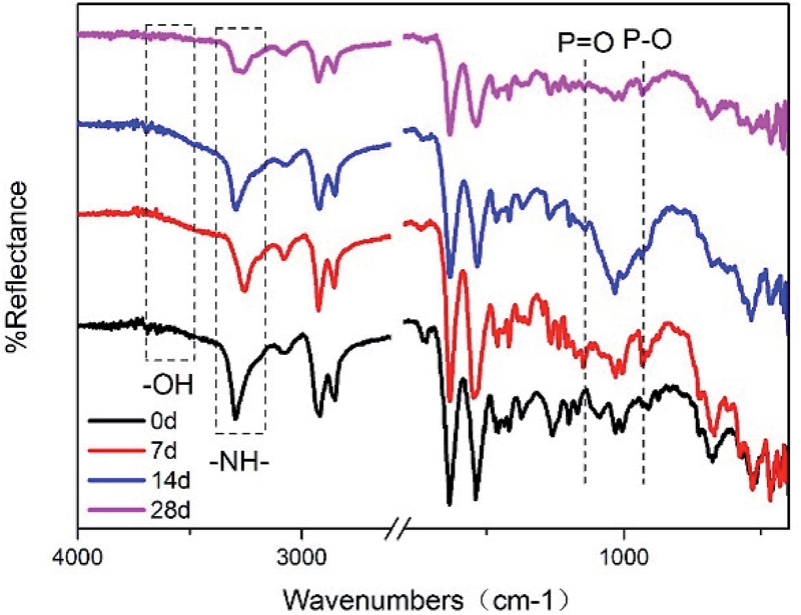
FTIR spectrum of environment tested connectors after different days.

Fig. 4 is an FTIR spectrum of the connectors after environmental testing. Compared with the initial connectors, the intensity of stretching vibration of –OH for the connectors environmentally tested was enhanced. The peaks representing stretching vibration of P–O and P=O were found at 931 cm^−1^ and 1143 cm^−1^,^22^ illustrated that the red phosphorus in the product released PH_3_ and then formed acids. At the same time, the generated phosphoric acid would accelerate the degradation of the polyamide. The peak representing –NH– shows significant changes, transferring to –NH_2_,^23^ especially for connectors tested by 28 days.

### Surface morphology

#### Scanning electron microscopy

**Fig. 5:**
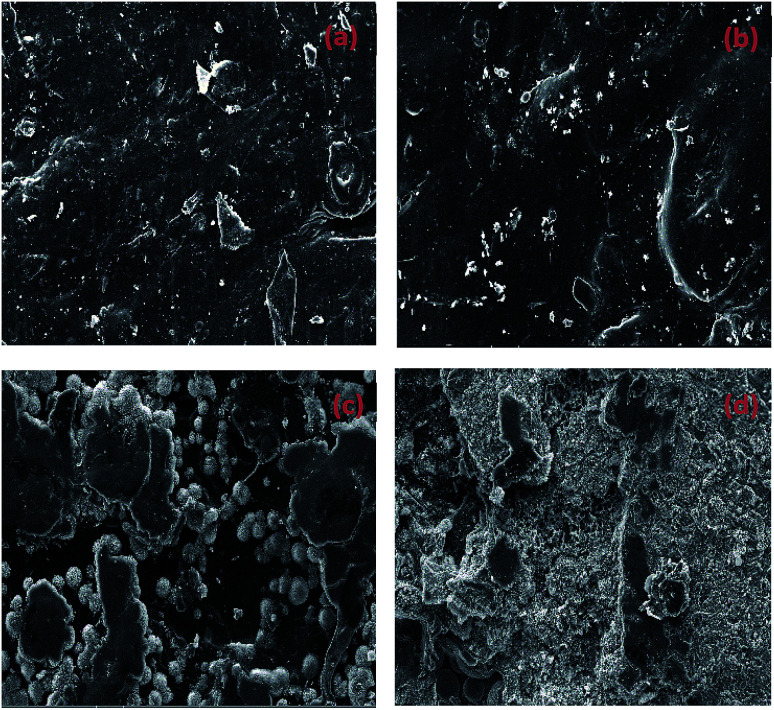
SEM images of connectors tested for 0 d (a), 7 d (b), 14 d (c), 28 d (d).

Fig. 5 is SEM images of connectors and its surface under 80 °C, 75% RH environment for 0 d, 7 d, 14 d and 28 d respectively. The surface of the original sample was relatively smooth. However, with the high temperature and humidity environment test progressing, cracks appeared on the surface, resulting in many flaky layers and increasing the roughness. Green globular substances appeared on the surface and section area of the sample after a period of time. It can be observed that the globular substance dispersed on the edge of the cracks after 14 days. After 28 days, the globes increased so much that it is too dense to observe a single sphere and the entire surface becomes shattered.

**Fig. 6:**
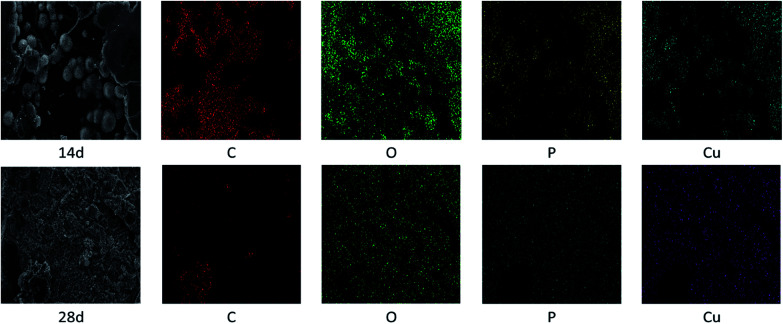
EDS images of connectors tested for 14 d and 28 d.

Fig. 6 and Table 2 exhibits the evolution of the elemental dispersion on the surface of connectors. As showed in Fig. 5, the spherical substance was mainly composed of P, O, and Cu elements. The elemental content given in Table 2 indicated that the substance was increasing over time. Phosphorus could not be detected on the surface at the beginning and after 7 days until the globular substance containing phosphorus was formed. With the time increase, the concentration gradient of phosphorus was produced from inside to surface. At the same time, the content of oxygen increased. It proved that phosphorus reacted with water and air to form phosphoric acid through these cracks in the high temperature and humidity environment and the phosphoric acid corroded the copper sheet attached to the connectors to form copper phosphate species on the surface.

**Table 2:** Surface elements content evolution from EDS

*T*/d	C/%	N/%	O/%	P/%	Cu/%
0	80.4	—	18.38	—	—
7	77.57	4.3	17.19	—	—
14	62.96	0.42	29.81	2.88	3.92
28	26.68	4.01	48.76	9.93	10.62

#### X-ray photoelectron energy spectrum analysis

**Fig. 7:**
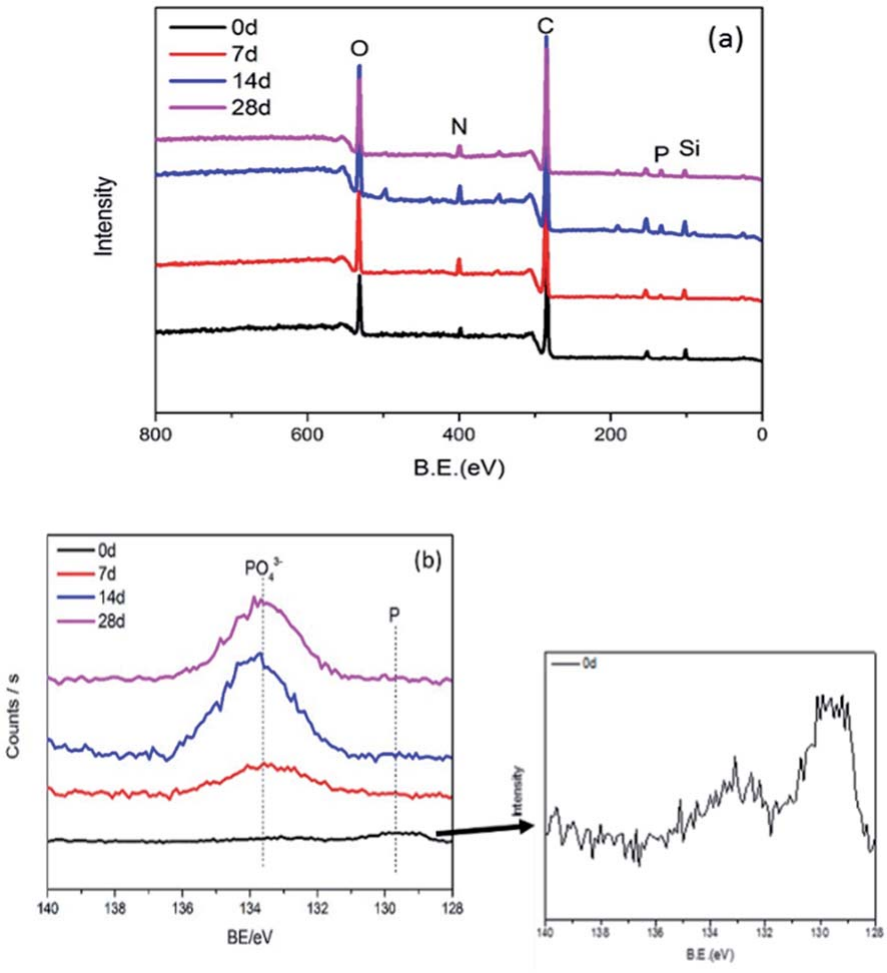
Wide-scan (a) and P 2p high-resolution (b) XPS spectra for connectors.

XPS spectrum of survey scan for connectors is exhibited in Fig. 7 and elemental content of connectors tested for different days is showed in Table 3. The intensity of carbon and oxygen is very strong with traces of N and Si existing. Nevertheless, the signal intensity of phosphorus is very low.

**Table 3:** Atomic content from survey scan of XPS

Relative atomic content/%	0 d	7 d	14 d	28 d
P/%	0.45	1.22	2.44	2.72
C/%	74.66	71.98	68.42	68.57
N/%	3.98	4.65	3.71	4.54
O/%	15.43	16.81	19.22	19.06
Ca/%	0.07	0.03	0.11	0.52
Cu/%	0	0.11	0.1	0.09
Si/%	5.41	5.19	5.99	4.5

The P 2p high-resolution spectra of connectors are exhibited in Fig. 7. There are two main peaks, 129.4 eV corresponding to red phosphorus; 133.4 eV corresponding to phosphate anion.^24^ The presence of these peaks affirms that red phosphorus was oxidized to phosphate. The intensities of peaks increases gradually and reaches a stable state, confirming that phosphate concentration increased with test prolonging, especially in the first 14 days.

In Table 3, the relative atomic content of five main elements P, C, N, O and Si, and traces of calcium were confirmed. In addition, due to the close contact between the connector and the copper sheet in the environmental test, a trace of copper was detected. As the experiment prolonged, the relative atomic concentration of phosphorus and oxygen on the surface of the product revealed an increasing tendency. This is consistent with the results of EDS. The red phosphorus flame retardant inside the connector migrated to the surface and was oxidized by air to form an acid. At the same time, the formed acid corroded the copper sheet to form copper phosphate, partially adhering to the product.

**Fig. 8:**
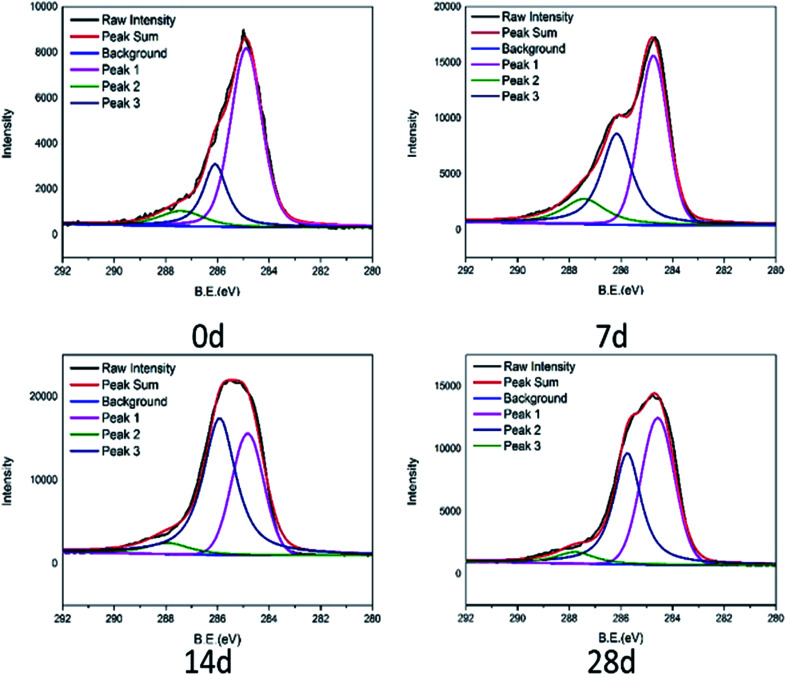
C 1s high-resolution XPS element spectra of connectors.

The C 1s spectra of tested connectors are fitted into three peaks to know the types of functional groups of PA66 from connectors in Fig. 8. The three peaks centered at 284.6 eV corresponding to C–C; 286.0–286.3 eV corresponding to C–O; 287.5–287.7 eV corresponding to C=O.^25^ While compared with the C 1s of the initial product, the intensity of the carbon–oxygen bond significantly increased. It is expected that PA66 was degraded and carbon–carbon bonds partially were broken, oxidized to carbon–oxygen bonds. The degradation of PA66 is a complicated process, mainly involving not only the hydrolysis of amide bonds, but also the breakage of molecular chains, accompanied by the formation of various small molecular compounds. The carbon–carbon bond is broken further, accelerating oxidization to cause the rise of the carbon–oxygen bond. This would generate cracks and further reduce the thermal stability of the connector, making it flammability.^26^

### Phosphate and phosphine analysis

In order to roughly determine the amount of the acid oxidized from red phosphorus, copper sheets was attached to connectors so that the acid could corrode them and transferred to the surface of copper sheets. The mass of phosphorus attached to copper was determined by inductively coupled plasma mass spectrometry (ICP-MS). Prior to this analysis, the copper pieces were digested in dilute hydrochloric acid to transfer the phosphorus corroding copper into the HCl solution. The results are shown in Table 4. After 80 °C/70% RH test for 7 days, the copper sheet was relatively less corrosive with only 8.294 μg of phosphorus detected. The copper sheets tested for 14 days and 21 days were corroded obviously and the weight of phosphorus reached 84.226 and 96.522 μg respectively. Furthermore, 169.66 μg phosphorus was detected after 28 days. As the test progresses, the phosphoric acid produced by red phosphorus continued to increase.

**Table 4:** Analysis of phosphorus content in corroded copper

*T*/d	7	14	21	28
Phosphorus weight/μg	8.294	84.226	96.522	169.66

Since the phosphine released by connectors reacted rapidly with water and air to form phosphoric acid, in order to determine the amount of phosphine, 2 gram connectors was placed in a dry gas sampling bag and tested by a phosphine detector. The results are shown in Table 5. Over time, the amount of phosphine released continued to increase.

**Table 5:** Content of phosphine released

*T*/d	7	14	21
Phosphine content/ppm	4.2	9.9	10.7

## Conclusions

**Fig. 9:**
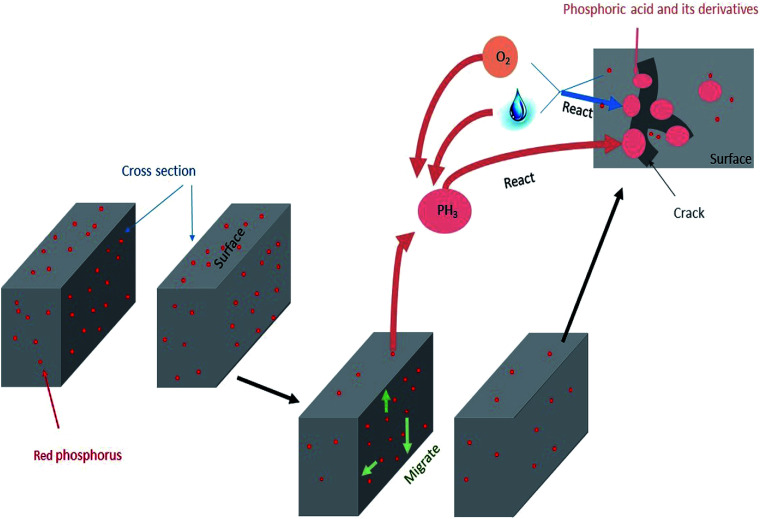
Failure behaviour mechanism diagram.

The mechanism of failure behaviour is revealed in Fig. 9. After connectors had undergone high temperature and humidity environmental testing, red phosphorus migrated to the surface and accumulated into several areas. At the same time, significant degradation occurred, the decomposition temperature was advanced and –OH and phosphorus oxides were formed. The surface morphology of connector became rough with cracks increasing and the copper attached to connector formed copper phosphate. The content of phosphorus and oxygen on the surface increased and the chemical environment of phosphorus changed from elemental phosphorus to phosphoric acid derivatives. The amount of phosphine released increased and it reacted with water and oxygen to form acids. Correspondingly, the formation of phosphoric acid derivatives accelerated the degradation process of nylon. Surface cracks caused by degradation resulted in red phosphorus to be further oxidized to an acid. The mutual promotion caused the failure behavior of the red phosphorus flame retardant PA66 electrical connector.

## Conflicts of interest

There are no conflicts to declare.

## Supplementary Material
